# Supplementary material for: Slik sculpts the plasma membrane into cytonemes to control cell-cell communication
Source: EMBO J. 2025 Mar 6;44(8):2186–210. doi: 10.1038/s44318-025-00401-8 (PMC12000455; doi:10.1038/s44318-025-00401-8)
Supplement: Supplementary file 2 — Movie EV1 [file 44318_2025_401_MOESM2_ESM.zip › Movie EV1.docx]

**Movie EV1:** Slik expression induces both stable and dynamic cytonemes. 2-min interval images from the apical-most region of wing disc DP cells expressing Slik-GFP showing stable (arrows) and dynamic (arrowheads) filopodia over 1h30. Scale bar = 10 µm.
