# Supplementary material for: Slik sculpts the plasma membrane into cytonemes to control cell-cell communication
Source: EMBO J. 2025 Mar 6;44(8):2186–210. doi: 10.1038/s44318-025-00401-8 (PMC12000455; doi:10.1038/s44318-025-00401-8)
Supplement: Supplementary file 3 — Movie EV2 [file 44318_2025_401_MOESM3_ESM.zip › Movie EV2.docx]

**Movie EV2:** Time lapse imaging of S2 cell expressing Slik^CCD^-GFP. Acquisition every 3 seconds for 10 minutes. The red square corresponds to the images represented in figure 4H, right and the kymograph. Scale bar = 10 µm.
