# Supplementary material for: Slik sculpts the plasma membrane into cytonemes to control cell-cell communication
Source: EMBO J. 2025 Mar 6;44(8):2186–210. doi: 10.1038/s44318-025-00401-8 (PMC12000455; doi:10.1038/s44318-025-00401-8)
Supplement: Supplementary file 4 — Movie EV3 [file 44318_2025_401_MOESM4_ESM.zip › Movie EV3.docx]

**Movie EV3:** dSTRIPAK depletion inhibits Slik-induced cytoneme formation. 4-min interval images showing side-by-side comparison of DP cells expressing Slik-GFP without (control, left) or with (right) Strip RNAi over 36 minutes. Scale bar = 10 µm.
