# Supplementary material for: Slik sculpts the plasma membrane into cytonemes to control cell-cell communication
Source: EMBO J. 2025 Mar 6;44(8):2186–210. doi: 10.1038/s44318-025-00401-8 (PMC12000455; doi:10.1038/s44318-025-00401-8)
Supplement: Supplementary file 12 — Expanded View Figures [file 44318_2025_401_MOESM12_ESM.pdf]

Expanded View Figures

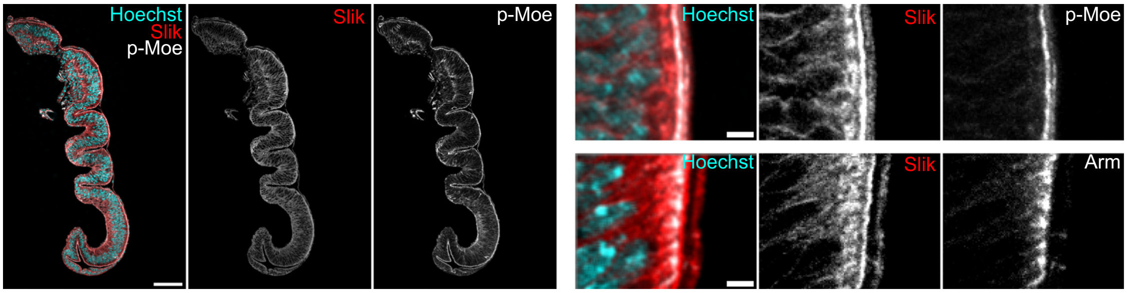

Figure EV1. Slik localizes to the apical-most free membrane in wild-type wing disc DP cells.

8 µm transverse section of w^{1118} wing disc prepared using cryosectioning protocol and immunostained for Slik (in red), phospho-Moesin (in white, left and top right) or Armadillo (β-catenin; in white, bottom right). Nuclei are stained with Hoechst (cyan). Scale bar = 50 µm (left) or 3 µm (right).

© The Author(s)

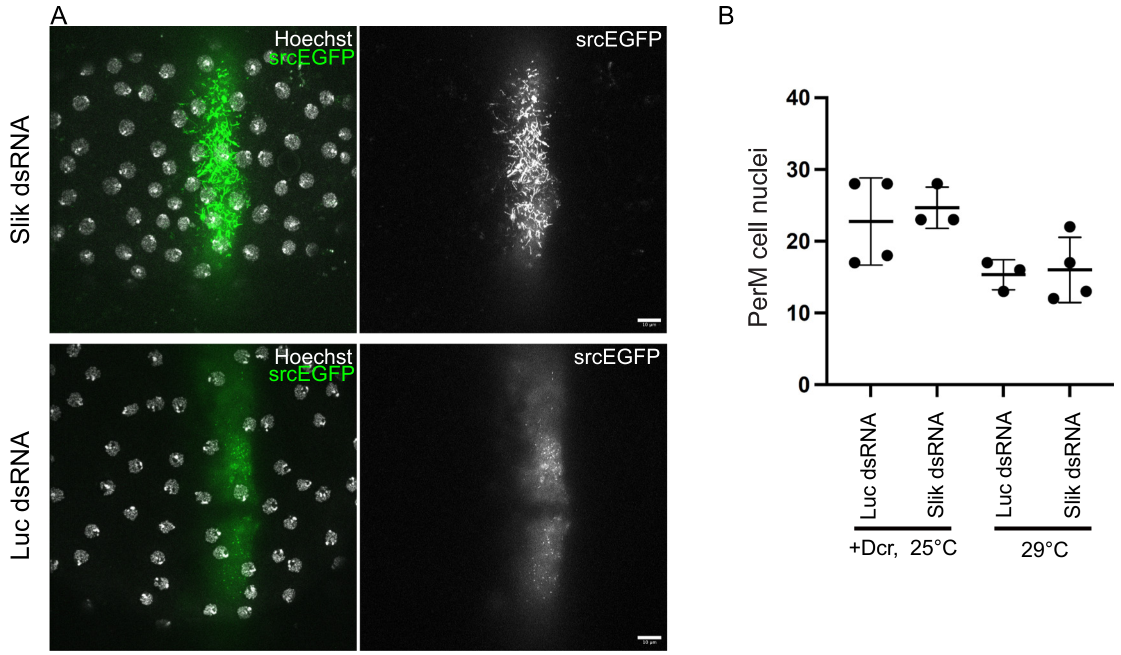

Figure EV2. Loss of Slik function induces nonfunctional filopodia.

(**A**) Projection of confocal sections through the PerM and apical-most region of the DP of discs expressing dsRNA targeting Slik (top) or Luciferase (control, bottom), together with the membrane marker srcEGFP (green), under the control of *ptc*-GAL4. Nuclei were stained with Hoechst (white). Scale bar = 10 µm. Slik depletion in srcEGFP-expressing DP cells caused the appearance of apical filopodia. (**B**) Quantification of the number of PerM cell nuclei in an 80 µm diameter circle above the DP expression domain of dsRNA targeting Slik or Luciferase, under the control of *ptc*-GAL4. The experiment was performed in two different conditions: with co-expression of Dicer (Dcr) at 25 °C (left), or at higher temperature (29 °C) without Dcr (right). Analysis was performed on (from left to right) 4, 3, 3, and 4 discs. Error bars indicate mean ± s.d. The filopodia formed upon Slik depletion did not support proliferative signaling.

© The Author(s)

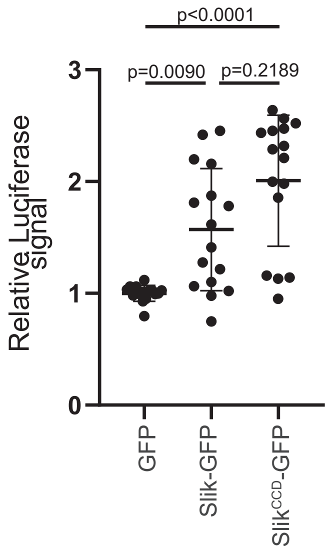

Figure EV3. Slik^{CCD}-dependent cytonemes are competent for cell–cell communication.

Co-culture experiment between donor cells expressing GFP, Slik-GFP or Slik^{CCD}-GFP and Hh and acceptor cells expressing Ci and Ptc-luciferase. GFP and Slik-GFP conditions were already shown in Fig. 2F. $n = 16$ from 4 independent experiments, each performed in quadriplicate. *P* values were calculated using Kruskal–Wallis test with Dunn's test for multiple comparisons. Source data are available online for this figure.

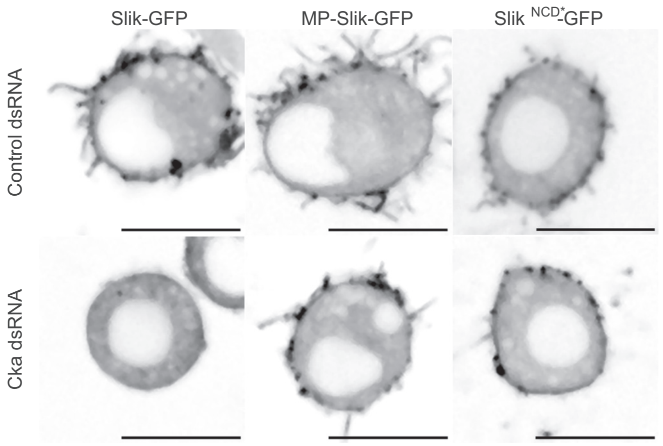

Figure EV4. MP-Slik-GFP and Slik^{NCD*}-GFP are still localized at the plasma membrane after Cka depletion.

Confocal microscopy images of cells expressing Slik-GFP, MP-Slik-GFP, or Slik^{NCD*}-GFP after treatment with dsRNA targeting Gal4 (Control, top row) or Cka (bottom row). Scale bars = 10 µm.

 © The Author(s)

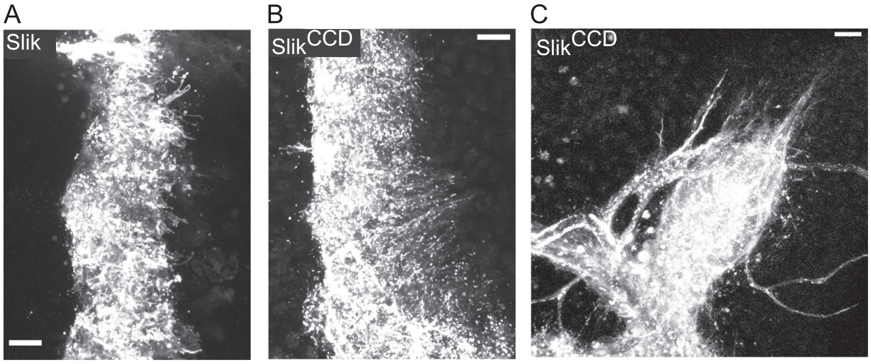

Figure EV5. Slik-GFP and Slik^{CCD}-GFP localize to cytonemes on the basal DP surface and on the air sac primordium.

(**A**, **B**) Projection of confocal sections through the basal region of the DP in discs expressing Slik-GFP (**A**) or Slik^{CCD}-GFP (**B**) under the control of *ptc*-GAL4. Slik localized to cytonemes emanating from the basal side of DP cells. Scale bars = 10 µm. (**C**) Projection of confocal sections through a wing sac primordium expressing Slik^{CCD}-mCherry under the control of *btl.S*-GAL4. Slik localized to cytonemes emanating from the air sac primordium. Scale bars = 10 µm.

© The Author(s)
